# Bracing Interventions Can Help Adolescents with Idiopathic Scoliosis with Surgical Indication: A Systematic Review

**DOI:** 10.3390/children9111672

**Published:** 2022-10-31

**Authors:** Fabio Zaina, Claudio Cordani, Sabrina Donzelli, Stefano Giuseppe Lazzarini, Chiara Arienti, Matteo Johann Del Furia, Stefano Negrini

**Affiliations:** 1 ISICO (Italian Scientific Spine Institute), 20141 Milan, Italy; 2 IRCCS Istituto Ortopedico Galeazzi, 20157 Milan, Italy; 3 IRCCS Fondazione Don Carlo Gnocchi, 20148 Milan, Italy; 4 Department of Biomedical, Surgical and Dental Sciences, University “La Statale”, 20122 Milan, Italy

**Keywords:** idiopathic scoliosis, brace, surgery, conservative treatment, rehabilitation, pediatrics, systematic review

## Abstract

There is a common agreement that bracing is appropriate for curves between 20 and 40° for the Cobb angle during growth, but for larger curves, the experts’ opinions are not consistent. We designed this systematic review to report the updated evidence about the effectiveness of bracing in scoliosis patients with curves ≥40° and a residual growth period. We included randomized controlled trials, non-randomized controlled trials, prospective and retrospective observational studies, and case series addressing the effect of bracing in patients with idiopathic scoliosis during growth with curves ≥40° for the Cobb angle, published from 2000 onwards. Outcome: The percentage of patients with surgery, curves above 45° or 50°, and a Cobb angle change are all included in the study. Nine papers (563 patients, average worst curve of 44.8°) are included: four are retrospective case series, two are retrospective and two are prospective cohort studies, and one is a prospective controlled study. The overall quality was good, with respect to the type of design. A total of 32% of the patients improved, 26% were stable, and 42% worsened. The rate of improvement ranged from 11% to 78%; the rate of worsening ranged from 4% to 64%. There are some studies suggesting the use of bracing even in the case of severe curves when patients are motivated by trying to avoid surgery. More and better-quality research with coherent outcome criteria is needed.

## 1. Introduction

Idiopathic scoliosis is a three-dimensional deformity of the trunk and spine that is usually diagnosed during growth [1]. Apart from negatively affecting the body appearance, it is generally asymptomatic in young patients but potentially a risk factor for back pain and a reduction in one’s quality of life (QoL) during adulthood. This condition has a variable trend to progression [2], derived from its entity, and to the growth phase, being more aggressive during rapid growth [3]. What happens during adulthood is dependent on the size of the curve at the end of the growth, with a high risk of progression for curves larger than a 50° Cobb angle [4]. While smaller curves are managed by observation, physiotherapeutic scoliosis specific exercises (PSSE), and bracing (soft, rigid, or superrigid [5]), for larger curves, surgery is frequently proposed [1]. A precise agreement about the threshold for surgery is inconclusive. The Scoliosis Research Society (SRS) states that bracing is appropriate for curves between 20 and 40°, while those with curves larger than 45° and a residual growth period should be surgically treated [6]. Some authors have a more aggressive approach, setting the threshold for surgery at 40° [7]. On the other side, the International Society on Scoliosis Orthopaedic and Rehabilitation Treatment (SOSORT) guidelines are more open to a conservative approach even in the case of large curves, suggesting a determinant role of the shared decision making [1]. The point is that the evidence for bracing is consistent for curves up to 40° [8], while there are SRS guidelines for brace studies which suggest avoiding bracing in the case of larger curves [9]. These guidelines have been updated by the SRS and SOSORT [10], but research on bracing surgical patients is still missing.

During the 1970s and 1980s, attempts to treat larger curves were made, but some authors found that results were inferior than the results generated in the case of smaller curves [11]. The attitude is gradually changing, with a more open approach to the role of bracing, since recent papers have suggested that bracing is not to be applied for curves larger than 60° [12], moving forward the threshold between a brace and surgery. Despite this new paper, experts’ opinions are not consistent on the topic, and the debate is still largely based on different perspectives more than the data. For these reasons, we designed this systematic review to report the current evidence about the effectiveness of bracing in scoliosis patients with curves larger than 40° and a residual growth period.

## 2. Materials and Methods

### 2.1. Design

A systematic review on bracing interventions on the Cobb angle modification in idiopathic scoliosis patients with surgical indication was performed. The review was conducted following the Preferred Reporting Items for Systematic Reviews and Meta Analyses (PRISMA) guidelines [13]. The protocol was registered on PROSPERO (CRD42022336905). 

### 2.2. Selection Criteria

#### 2.2.1. Type of Study

We included randomized controlled trials (RCTs), non-randomized controlled trials (NRCTs), prospective and retrospective observational studies, as well as case series addressing the effect of bracing interventions in people with idiopathic scoliosis with curves ≥ a 40° Cobb angle which were published from 2000 onwards. 

#### 2.2.2. Population

The study population included idiopathic scoliosis patients below 18 years of age who were treated with a brace intervention with a Cobb angle equal to or larger than 40°.

#### 2.2.3. Interventions

We included studies addressing bracing interventions alone or combined with other conservative treatments aimed at modifying the Cobb angle for people with idiopathic scoliosis. Drugs and surgical interventions were not considered eligible and were consequently excluded.

#### 2.2.4. Comparator(s)

We included studies that compared the bracing interventions with any other type of intervention, no intervention, exercise, or surgical interventions. Due to the inclusion of case series, we also considered those with no comparison.

#### 2.2.5. Outcomes

We considered the Cobb angle changes at the end of the treatment:

- The average worst Cobb angle before and after treatment;
- The percentage of patients with improvements (reduction in the Cobb angle >5°), progression (increase >5°), or stability (±5°);
- The percentage of patients with curves larger than 45°;
- The percentage of patients with curves larger than 50°;
- The percentage of surgically treated patients.

### 2.3. The Search Strategy and Screening

The search was performed by an information specialist (SGL) on 9 May 2022, in the following databases: PubMed, EMBASE, the Cumulative Index to Nursing & Allied Health Literature (CINAHL), and Cochrane Central Register of Controlled Trials (CENTRAL) using the following keywords: scoliosis, spinal diseases, orthotic devices, and brace (see the Appendix A for the full search strategy). Moreover, the records of the SOSORT meetings’ abstracts were screened.

Four review authors (CA; CC; FZ; and SD) screened the title abstracts and full-text articles, with the resolution of any conflicts being conducted by another review author (SN). We excluded the case reports and cross-sectional studies of individuals. We also excluded secondary analyses, economic analyses, or poster abstracts only, expert opinions, letters to the editor, non-English full text or the full text not being available, and all the studies that did not match the inclusion criteria. For papers retrieved in abstract books, the authors were contacted, and all the needed data were required.

### 2.4. Assessment of Risk of Bias and Critical Appraisal in Included Studies

One author (CC) applied Cochrane’s ‘risk of bias’ tool to the included RCT studies for each outcome, and a second verified his judgments (CA). The disagreements were solved by a consensus or by a consultation with a third review author (SN).

We assessed the risk of bias in the included RCTs using the Cochrane’s ‘risk of bias’ tool, described in the Cochrane Handbook for Systematic Reviews of Interventions [14]. We assessed the following domains: the allocation sequence generation (selection bias), allocation concealment (selection bias), blinding of participants (performance bias), blinding of personnel or care providers (performance bias), blinding of objective outcome assessment (detection bias), blinding of subjective outcome assessor (detection bias), incomplete outcome data (attrition bias), selective reporting (reporting bias), group similarity at the baseline (other bias-selection bias), and intention to treat analysis (other bias). Each domain of the studies was classified to have a ‘low risk’, ‘high risk’, or an ‘unclear risk’, and we evaluated the bias of the individual items as described in the Cochrane Handbook for Systematic Reviews of Interventions [14].

However, for NRCTs and analytical observational studies, we used the JBI critical appraisal tools [15]. 

Our objective was to provide an answer to the following questioning:

- NRCTs: if it is clear in the study what is the ‘cause’ and what is the ‘effect’; if the participants included in any comparisons were similar; if the participants included in any comparisons received a similar treatment/care, other than the exposure or intervention of interest; if there was a control group; if there were multiple measurements of the outcome both pre- and post-intervention/exposure; if the follow-up was complete and, if not, if differences between the groups in terms of their follow-up were adequately described and analyzed; if the outcomes of the participants included in any comparisons were measured in the same way; if the outcomes were measured in a reliable way; and finally if an appropriate statistical analysis was used.
- Observational studies: if the two groups were similar and recruited from the same population; if the exposures were measured similarly to assign people to both the exposed and unexposed groups; if the exposure was measured in a valid and reliable way; if confounding factors were identified; if strategies to deal with the confounding factors were stated; if the groups/participants were free of the outcome at the start of the study (or at the moment of exposure); if the outcomes were measured in a valid and reliable way; if the follow-up time was reported and sufficient to be long enough for the outcomes to occur; if the follow-up was complete, and if not, were the reasons for the lack of a follow-up described and explored; if strategies to address the incomplete follow-up were utilized; and if the appropriate statistical analysis was used?
- Case series: if there were clear criteria for the participants’ inclusion; if the condition was measured in a standard, reliable way for all the participants included; if valid methods were used for the identification of the condition for all the participants included; if the case series had a consecutive inclusion of the participants; if the case series had completed the inclusion of participants; if there was a clear reporting of the demographics of the participants; if there was a clear reporting of the clinical information of the participants; if the outcomes or a follow-up to the results were clearly reported; if there was a clear reporting of the presenting site(s)/clinic(s) demographic information; and if the statistical analysis was appropriate.

Each question of the studies was answered as ‘yes’, ‘no’, or ‘unclear/not applicable’.

### 2.5. Summary of Findings and Assessment of the Certainty of the Evidence

Three review authors (FZ, CC, and MJDF) extracted the data on the study characteristics using Microsoft Excel before comparing the findings. We used a predetermined data form to extract the features of the included papers, including the:

1. Report characteristics (year, author, title, DOI, country, and aim).
2. Study design (groups and the number of participants).
3. Characteristics of the intervention (description of the intervention, frequency, and duration).
4. Comparator characteristics (description of intervention, frequency, and duration).
5. Outcomes assessed and measures (type of outcomes and how outcomes are measured).
6. Numerical data for the outcomes of interest (effect size between groups, statistically significance).

We solved any differences in opinion about the study characteristics and methodological limitations of the studies by a consensus with a second review author (SD).

## 3. Results

**Figure 1:**
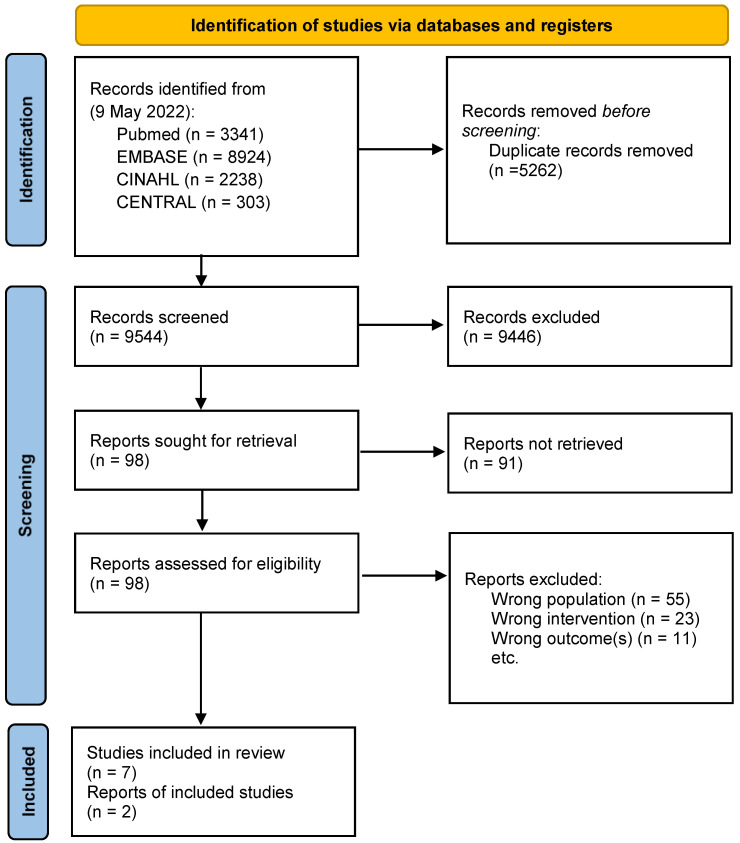
PRISMA Diagram.

Nine thousand five hundred and forty-five titles were screened, from which 98 papers were retrieved and 9 were finally included (Figure 1). Four were retrospective case series [16,17,18,19], two were retrospective cohort studies [20,21], two were prospective cohort studies [22,23], and one was a prospective controlled study [24]. A total of 563 idiopathic scoliosis patients were included with an average worst curve of 44.9 ± 4.2° Cobb angle (Table 1). 

### Assessment of Risk of Bias and Critical Appraisal in Included Studies

In case series [16,17] all questions on the JBI checklist received a positive answer, except for the clear reporting of the demographical characteristics of the participants. In regard to the observational studies [20,24], the main methodological limitations were associated with the absence of a confounding factors identification and the strategies for managing them, as well as the application of strategies to address the incomplete follow-up visits. Finally, the study assessed as an NRCT [23] demonstrated the main methodological shortcoming in the absence of a control group. Table 2 provides the results of the critical appraisal performed on five of the nine studies included in the present review.

Due to the characteristics of the studies, a meta-analysis was not performed. Results have been reported narratively, including the tabulation of the data and a summary of the evidence (Table 1 and Table 3).

Considering the whole sample of studies for which we have complete data (563 patients), 32% of the patients improved (>5° Cobb change), 26% were stable, and 42% progressed. The rate of improvement ranged from 11% to 78%, while the rate of progression ranged from 4% to 64%. The surgical rates were not reported in all studies, but ranged from 0 to 58%, with relevant differences in different studies.

In three studies [20,23,24], the number of patients with curves below 45° largely increased after treatment. In one paper, the percentage of patients with curves larger than 45° increased [16] while in another, those larger than 50° increased [21].

Considering the retrospective studies (332 patients, 44.4° ± 3.5° Cobb angle at the baseline), 18.4% of patients improved (>5° Cobb angle change), 30.1% were stable, and 51.5% worsened. For the prospective studies, both a per-protocol analysis and an intention-to-treat (ITT) are available. For the per-protocol (198 patients, 47.8° ± 5.2° Cobb angle at the baseline), 61.9% of patients improved, 22.9% remained stable, and 15% progressed while for the ITT, (171 patients, 48.2° ± 5.2° at the baseline) 59% improved, 11% remained stable, and 29% progressed (Table 3).

## 4. Discussion

Large scoliosis is a challenge for a treating physician because of the high risk of surgery. As this procedure is invasive, risky, and based on the loss of spinal movement, one of the main goals of conservative treatment should be to try to avoid the need for surgery [1]. Bracing is the most effective conservative approach, generally accepted and applied in curves with up to a 40° Cobb angle [6]. The findings of this review show that it is also possible to obtain good results, including improvements, in the case of larger curves. 

The results of the current review are based on studies with low-quality designs: we did not retrieve any RCTs, and only one study included a control group. Nevertheless, considering the quality criteria of each specific design, most of the studies were well performed. Unfortunately, in this field, it is almost impossible to run randomized clinical trials since patients refuse randomization. This was already demonstrated by different authors and their failed attempts [25,26]. Thus, we can assume that the best evidence will come from good-quality prospective controlled studies. Another limitation of the current literature is the absence of coherence in the reporting results: specific criteria would be needed, specifically for less severe curves [9,10]. The SOSORT guidelines [4] suggest the main aim of conservative treatment is avoiding surgery, and this outcome should always be reported either in terms of a real avoidance, or better in terms of the number of patients who reached a significant threshold, whether below 45° according to the SRS criteria [9], or below 50° according to the BrAIST study [26]. Moreover, it is important to remember that in these scoliosis patients with more severe curves, the lesser the curve, the lower the risks of problems in adulthood [3]. For this reason, the commonly reported percentage of improvement is also essential.

It is a traditional and common idea that bracing can only prevent the progression of the deformity with no chance of improving, with a progressive reduction in the efficacy as the curve size increases [27]. The data collected in this review shows that even in the case of large surgical curves, it is possible to partially correct the deformity or at least stop the progression. In all but one of the included papers [19], for which the data are missing, there is a group of improved patients. This shows the potential for a brace treatment in patients who are willing to avoid surgery. A threshold of 40° for a surgical indication has been established from well-known surgeons and researchers in the field [7], while others set it higher at 45° [6]. In our perspective, the threshold should be increased, as proposed by other recent papers that stated that bracing is not appropriate for curves larger than 60° [12]. Moreover, there is no doubt that the there is a grey area where the options of bracing and surgery overlap, and that an evidence-based clinical approach requires an informed shared decision making. The commitment of the patient is certainly a pillar of conservative scoliosis treatment. All these papers included patients who refused surgery and wanted to be treated with a brace. With this premise, it is reasonable to try a bracing approach, also considering that in about 6 months it is possible to have a quite reliable prediction of the final results [28], thus allowing to go on with even more motivation or eventually shift to surgery. Despite a common trend, there were differences in the results of the treatment. There can be several explanations for this: the different braces, the daily dosage, whether PSSE was performed or not, the age at the start of treatment, and the skeletal maturity. All these factors play a role, but we cannot rule out which is which because of an incomplete reporting and differences in the included population. Moreover, this goes beyond the scope of this paper.

In this review, we wanted to focus on recent papers trying to address the issue of severe curves to be treated during growth because braces are changing over time, so we chose to report the most updated scenario from 2000 onwards. Even before, other authors have tried to manage curves larger than 40° with bracing, but they generally concluded that the effect in large curves was inferior with respect to the smallest ones [11]. In a group of patients treated with a Boston brace, Emans reported an average improvement from 41.8° to 34.6° in curves originally between 40 and 49°, and from 52.7° to 48.0° in curves between 50° and 59° [29]. In a group of patients treated with a Milwaukee brace, Edmonson reported, for lumbar curves, a stability for seven patients who started with a Cobb angle between 40° and 49°, and an average improvement of 9° for three patients with curves larger than 50°. For thoracic curves, 5 patients improved on average by 4° and 10 patients improved by 11° [30].

The results presented by some of the papers reported in this review are quite good. Nevertheless, some authors seem to interpret their results as not being good enough, thus suggesting to avoid bracing larger curves. This is probably due to a more limited knowledge of the natural history of scoliosis that we had in the past. The findings of the untreated arm of the BrAIST study surprised many experts since it showed the aggressiveness of the smallest curves to be much higher than expected [26], and this is probably also true for larger curves.

The present paper has some limitations. First, the quality of the evidence is very low due to the design and the small samples of the included papers. Nevertheless, all the papers were consistent in reporting not just the stability but even the improvements in the worst curves. The endpoint for the included studies was the end of treatment. A long-term follow-up is missing, but we can refer to other papers reporting over time regarding what can influence the stability or further progression during adulthood, even after a brace treatment, to be mainly the size of the curve at the end of growth [31]. It was not possible to perform a metanalysis because of the small number and the characteristics of the included papers. The interventions which were used varied across studies, including different braces in terms of the design, material, biomechanical principles, and treatment protocol [5]. The heterogeneity of the interventions is paired to the heterogeneity of the outcome measures. This heterogeneity exposes the risk of misleading results from a metanalysis. Moreover, the absence of a control group in all but one study allows only a descriptive synthesis through the weighted means. 

The included papers reported patients who refused surgery, so we can assume they were very motivated for the treatment, thus limiting the generalizability of the results. Brace treatment can be very tough on many youngsters and multiple studies on their quality of life in the long-term suggest that operated patients are more satisfied with the management of their condition than brace-treated individuals, even if the quality of life during adulthood is similar between operated and braced patients [32]. Nevertheless, evidence from the highest-quality studies questions the traditionally reported negative impact of bracing, reporting that observed and treated patients showed a similar quality of life [33]. There are also reports showing that, in cases where the same brace is applied, the impact of the treatment is more correlated to the support of the treating team that the brace itself [34]. Moreover, surgery has the relevant drawback of limiting spinal movement and it is associated with the relevant risks and side effects that occur in 5 to 23% of procedures [35]. 

## 5. Conclusions

According to the findings of this systematic review, we can conclude that there is very low-quality evidence supporting the use of bracing in severe curves, when patients are motivated and willing to avoid surgery. Nevertheless, we need more research with coherent outcome criteria. From a clinical standpoint, the advantages and limitations of bracing in such a condition should be discussed with the patients and family, compared with those of surgery, with the aim of reaching a shared decision. Further and better high-quality studies are needed to rule out the current limitations, as well as to explore which brace, and which protocols of treatment, are more effective for patients with severe scoliosis.

## Figures and Tables

**Table 1:** Characteristic of the included studies.

Brace		N	Age		Sex (%F)	Risser		Baseline Cobb				Study	
												Design	Ref
			Av	±		Av	±	Av	±	Min	Max		
Boston		100	11.8	2.36	85	0.8	1.2	45	3.9	40	60	RCS	Verhofste et al., 2020 [16]
Boston		90	12.6	1.3	84	1	1.2	42.5	2.1	40	45	RCS	Xu et al., 2019 [17]
Boston		54	13.7		83			43.4	2.4			RCS	Zhu et al., 2017 [18]
Milwaukee													
Lion		28	14.2		86			49.4	3.8	45	48	RC	Negrini et al., 2011 [19]
Risser cast													
Sforzesco													
Milwaukee		60	12.6		88			44.9	4.8			RC	Razeghinezhad et al., 2021 [20]
Cheneau		48	12.3		98			47	5.3			PC	Karavitas et al., 2022 * [21]
Rigo Cheneau		12			100			45		41	50	PCS	Maruyama et al., 2014 * [22]
Lion	ITT	132						47	5.3			PC	Aulisa et al., 2018 [23]
PASB	EA	104	12.9		93			47	5.3			PC	Aulisa et al., 2018 [23]
Sforzesco	ITT	39	15.3					52.5		45	93	PCC	Lusini et al., 2013 [24]
	EA	34			81			52.5		45	93	PCC	Lusini et al., 2013 [24]
**Weighted means**													
Total		563	12.6	1.8	94	1.0	1.2	44.9	4.2				
Retrospective studies		332	12.7	1.8	85	1.1	1.2	44.4	3.4				
Prospective studies EA		198	10.5	1.8	93	0.7	0.8	47.8	5.3				
Prospective studies ITT		171	15	1.1		2		48.3	5.3				

Abbreviations: + improved; = stable; − progressed. Av = average; ± standard deviation; Min = minimum; Max = maximum; Ref = reference; F = female; EA = efficacy analysis; ITT = intention to treat; RCS = retrospective case series; RC = retrospective cohort; PC = prospective cohort; PCS = prospective case series; PCC = prospective controlled cohort. * not published.

**Table 2:** Critical appraisal of the published studies included in the review.

**Study** **(Case Series)**	**1**	**2**	**3**	**4**	**5**	**6**	**7**	**8**	**9**	**10**
Verhofste 2020 [16]	+	+	+	+	+	−	+	+	+	+
Xu 2019 [17]	+	+	+	+	+	−	+	+	+	+
**Study (observational studies)**	**1**	**2**	**3**	**4**	**5**	**6**	**7**	**8**	**9**	**10**
Negrini 2011 [19]	+	+	+	−	−	+	+	+	−	−
**Study** **(Non-randomized Controlled Trials)**	**1**	**2**	**3**	**4**	**5**	**6**	**7**	**8**	**9**	
Lusini 2013 [24]	+	+	+	+	+	+	+	+	+	
Aulisa 2018 [23]	+	+	+	−	+	+	+	+	+	

**Abbreviations:** NRCT = non-randomized controlled trial; + yes; − no. **Case series items**: (1) were there clear criteria for inclusion in the case series? (2) Was the condition measured in a standard, reliable way for all participants included in the case series? (3) Were valid methods used for the identification of the condition for all participants included in the case series? (4) Did the case series have a consecutive inclusion of the participants? (5) Did the case series have complete the inclusion of the participants? (6) Was there a clear reporting of the demographics of the participants in the study? (7) Was there a clear reporting of the clinical information of the participants? (8) Were the outcomes or follow-up results of cases clearly reported? (9) Was there a clear reporting of the presenting site(s)/clinic(s) demographic information? 10) Was statistical analysis appropriate? **Observational studies items**: (1) were the two groups similar and recruited from the same population? (2) Were the exposures measured similarly to assign people to both exposed and unexposed groups? (3) Was the exposure measured in a valid and reliable way? (4) Were the confounding factors identified? (5) Were strategies to deal with the confounding factors stated? 6) Were the groups/participants free of the outcome at the start of the study (or at the moment of exposure)? (7) Were the outcomes measured in a valid and reliable way? (8) Was the follow-up time reported and sufficient to be long enough for outcomes to occur? (9) Was a follow-up complete, and if not, were the reasons for a lack of a follow-up described and explored? (10) Were the strategies to address the incomplete follow-up utilized? **NRCT items**: (1) is it clear in the study what is the ‘cause’ and what is the ‘effect’? (2) Were the participants included in any comparisons similar? (3) Were the participants included in any comparisons receiving a similar treatment/care, other than the exposure or intervention of interest? (4) Was there a control group? (5) Were there multiple measurements of the outcome both pre- and post-intervention/exposure? (6) Was the follow-up complete and, if not, were the differences between the groups in terms of their follow-up adequately described and analyzed? (7) Were the outcomes of the participants included in any comparisons measured in the same way? (8) Were the outcomes measured in a reliable way? (9) Was the appropriate statistical analysis used?

**Table 3:** Data provided by each included study on bracing effects.

Brace		Cobb Angle Change (%)					Curves ≥ 45° (%)		Curves ≥ 50° (%)		Surgery (%)	Treatment Duration (Years)	Study
							Pre	Post	Pre	Post			
		+	=	−	Av	±							
Boston		11	32	57	8	14.8	46	74	13		58	1.8 (1.2–1.9) *	Verhofste et al., 2020
Boston		13.3	37.8	48.9	−0.4	8.9						2.4 (1.3)	Xu et al., 2019
Boston		14	22	64	1.8	9.8					0	2.3 (1.8-2.6) **	Zhu et al., 2017
Milwaukee													
Lion		71	25	4	−9.25	8.04	100	32	43	7	0	4 (1.5-7.4) **	Negrini et al., 2011
Risser cast													
Sforzesco													
Milwaukee		18	25	57	2.36	11.6			13	40	52	3.1 (1.7)	Razeghinezhad et al., 2021
Cheneau		27.1	50	22.9	Th: −2.5		77	60	54	44		3	Karavitas et al., 2022
					L: −5.2								
Rigo Cheneau		0	50		5.3		42		0	50	25	3	Maruyama et al., 2014
Lion	ITT	61	11	28								5.3 (1.5)	Aulisa et al., 2018
PASB	EA	78	13	9	−12.83	9.9	55	21			15 §		Aulisa et al., 2018
Sforzesco	ITT	54	13	33			100	38.5			15 ^	5.3 (1.1)	Lusini et al., 2013
	EA	62	15	23	−10.4	10.7							Lusini et al., 2013
**Weighted means**													
Total		31	27	42	−1.83		64	47	24	36	29		
Retrospective studies		18	30	51.5	1.81		58	65	17	30			
Prospective studies EA		58	25	17	−11.25		50	33	43	45			
Prospective studies ITT		59	11	29			55	39					

Abbreviations: Av = average; ± = standard deviation; + improved; = stable; − worsened; ITT = intention to treat; EA = efficacy analysis; Th = thoracic; L = lumbar. § surgery referral; ^ fused (0%) or in waiting list for fusion (15%); * data reported as median and interquartile range; and ** data reported as median and minimum/maximum range.

## Data Availability

All included data come for published papers or studies presented at scientific meetings.

## Appendix A. Search Strategies

**PubMed (via pubmed-ncbi.nlm.nih.gov/ (accessed on 9 May 2022))**
1. “Scoliosis”[MeSH Terms] OR “Spinal Diseases”[MeSH Terms] 134,975 results 2. “Spinal disease*”[Title/Abstract] OR “Scoliosis*”[Title/Abstract] OR “idiopathic* scoliosis”[Title/Abstract] 26,357 results 3. #1 OR #2 142599 results 4. “Orthotic Devices”[MeSH Terms] OR “Braces”[MeSH Terms] 16,212 results 5. “brace*”[Title/Abstract] OR “bracing”[Title/Abstract] OR “braces treat*”[Title/Abstract] OR “orthopedic equip*[Title/abstract]12,005 results 6. #4 OR #5 23,311 results 7. #3 AND #6 3341 results (((“spinal diseases”[MeSH Terms]) OR (“spinal disease*”[Title/Abstract]) OR (“scoliosis*”[Title/Abstract]) OR (“idiopathic scoliosis”[Title/Abstract])) AND ((“orthotic devices”[MeSH Terms]) OR (“braces”[MeSH Terms]) OR (“brace*”[Title/Abstract]) OR (“brace treat*”[Title/Abstract]) OR (“bracing”[Title/Abstract])))
**Embase (via Embase.com (accessed on 9 May 2022))**
1. (‘spine’/exp OR ‘spine disease’/exp) 424,813 results 2. (‘idiopathic* scoliosis’:ti,ab,kw OR ‘spinal disease*’:ti,ab,kw OR ‘scoliosis*’:ti,ab,kw) 34,587 results 3. #1 OR #2 427606 results 4. (‘orthosis’/exp OR exp OR ‘brace’/exp) 38,200 results 5. (‘brace*’:ti,ab,kw OR ‘bracing’:ti,ab,kw) 13,784 results 6. #4 OR #5 44,170 results 7. #3 AND #6 8924 results (((‘spine’/exp OR ‘spine disease’/exp) OR (‘idiopathic* scoliosis’:ti,ab,kw OR ‘spinal disease*’:ti,ab,kw OR ‘scoliosis*’:ti,ab,kw)) AND ((‘orthosis’/exp OR ‘brace’/exp) OR (‘brace*’:ti,ab,kw OR ‘bracing’:ti,ab,kw)))
**CINAHL (via EBSCOhost (accessed on 9 May 2022))**
1. (MH “Spine+”) OR (“Spinal Diseases+”) OR (MH “Scoliosis”) 71,860 results 2. TI ((“scoliosis*” OR (idiopathic* W1 scoliosis) OR (spinal W1 disease*)) OR AB ((“scoliosis*” OR (idiopathic* W1 scoliosis) OR (spinal W1 disease*)) OR SU ((“scoliosis*” OR (idiopathic* W1 scoliosis) OR (spinal W1 disease*)) 19,967 results 3. #1 OR #2 74941 results 4. (“Orthoses+”) 11,272 results 5. TI ((“orthos*” OR “brace*” OR “bracing”)) OR AB ((“orthos*” OR “brace*” OR “bracing”)) OR SU ((“orthos*” OR “brace*” OR “bracing”)) 18,426 results 6. #4 OR #5 19,122 results 7. #3 AND #6 2238 results ((MH “Spine+”) OR (“Spinal Diseases+”) OR (MH “Scoliosis”)) OR (TI ((“scoliosis*” OR (idiopathic* W1 scoliosis) OR (spinal W1 disease*)) OR AB ((“scoliosis*” OR (idiopathic* W1 scoliosis) OR (spinal W1 disease*)) OR SU ((“scoliosis*” OR (idiopathic* W1 scoliosis) OR (spinal W1 disease*))) AND ((“Orthoses+”) OR TI (“orthos*” OR “brace*” OR “bracing”) OR AB (“orthos*” OR “brace*” OR “bracing”) OR SU (“orthos*” OR “brace*” OR “bracing”))
**CENTRAL (Via Cochrane Library)**
1. MeSH descriptor: [Scoliosis] explode all trees 473 results 2. MeSH descriptor: [Spine] this term only 754 results 3. “scoliosis*”:ti,ab,kw 1437 results 4. #1 OR #2 OR #3 2161 results 5. MeSH descriptor: [Braces] explode all trees 442 results 6. MeSH descriptor: [Orthotic Devices] this term only 603 results 7. #5 OR #6 1030 results 8. “brace*”:ti,ab,kw 1979 results 9. “bracing”:ti,ab,kw 597 results 10. “Orthotic NEAR/1 device*”:ti,ab,kw 672 results 11. #8 OR #9 OR #10 2785 results 12. #5 AND #11 303 results

## References

[1] Negrini S., Donzelli S., Aulisa A.G., Czaprowski D., Schreiber S., De Mauroy J.C., Diers H., Grivas T.B., Knott P., Kotwicki T. (2018). 2016 SOSORT guidelines: Orthopaedic and rehabilitation treatment of idiopathic scoliosis during growth. Scoliosis Spinal Disord..

[2] Di Felice F., Zaina F., Donzelli S., Negrini S. (2018). The Natural History of Idiopathic Scoliosis during Growth: A Meta-Analysis. Am. J. Phys. Med. Rehabil..

[3] Hresko M.T. (2013). Clinical practice. Idiopathic Scoliosis in Adolescents. N. Engl. J. Med..

[4] Weinstein S.L. (2019). The Natural History of Adolescent Idiopathic Scoliosis. J. Pediatr. Orthop..

[5] Negrini S., Aulisa A.G., Cerny P., de Mauroy J.C., McAviney J., Mills A., Donzelli S., Grivas T.B., Hresko M.T., Kotwicki T. (2022). The classification of scoliosis braces developed by SOSORT with SRS, ISPO, and POSNA and approved by ESPRM. Eur. Spine J..

[6] Adolescent Idiopathic Scoliosis | Scoliosis Research Society. https://www.srs.org/professionals/online-education-and-resources/conditions-and-treatments/adolescent-idiopathic-scoliosis.

[7] De Kleuver M., Lewis S.J., Germscheid N.M., Kamper S.J., Alanay A., Berven S.H., Cheung K.M., Ito M., Lenke L.G., Polly D.W. (2014). Optimal surgical care for adolescent idiopathic scoliosis: An international consensus. Eur. Spine J..

[8] Negrini S., Minozzi S., Bettany-Saltikov J., Chockalingam N., Grivas T.B., Kotwicki T., Maruyama T., Romano M., Zaina F. (2015). Braces for idiopathic scoliosis in adolescents. Cochrane Database Syst. Rev..

[9] Richards B.S., Bernstein R.M., D’Amato C.R., Thompson G.H. (2005). Standardization of Criteria for Adolescent Idiopathic Scoliosis Brace Studies: SRS Committee on Bracing and Nonoperative Management. Spine.

[10] Negrini S., Boards S., Hresko T.M., O’Brien J.P., Price N., SOSORT Boards, SRS Non-Operative Committee Recommendations for Research Studies on Treatment of Idiopathic Scoliosis (2015). Consensus 2014 between SOSORT and SRS non–operative management committee. Scoliosis.

[11] Carr W.A., Moe J.H., Winter R.B., Lonstein J.E. (1980). Treatment of idiopathic scoliosis in the Milwaukee brace. J. Bone Jt. Surg..

[12] Roye B.D., Simhon M.E., Matsumoto H., Bakarania P., Berdishevsky H., Dolan L.A., Grimes K., Grivas T.B., Hresko M.T., Karol L.A. (2020). Establishing consensus on the best practice guidelines for the use of bracing in adolescent idiopathic scoliosis. Spine Deform..

[13] Page M.J., McKenzie J.E., Bossuyt P.M., Boutron I., Hoffmann T.C., Mulrow C.D., Shamseer L., Tetzlaff J.M., Akl E.A., Brennan S.E. (2021). The PRISMA 2020 Statement: An Updated Guideline for Reporting Systematic Reviews. BMJ.

[14] Akl E., Altman D., Aluko P., Askie L., Beaton D., Berlin J., Bhaumik B., Bingham C., Boers M., Booth A. (2019). Cochrane Handbook for Systematic Reviews of Interventions.

[15] Critical Appraisal Tools | JBI. https://jbi.global/critical-appraisal-tools.

[16] Verhofste B.P., Whitaker A.T., Glotzbecker M.P., Miller P.E., Karlin L.I., Hedequist D.J., Emans J.B., Hresko M.T. (2020). Efficacy of bracing in skeletally immature patients with moderate–severe idiopathic scoliosis curves between 40° and 60°. Spine Deform..

[17] Xu L., Yang X., Wang Y., Wu Z., Xia C., Qiu Y., Zhu Z. (2019). Brace Treatment in Adolescent Idiopathic Scoliosis Patients with Curve between 40° and 45°: Effectiveness and Related Factors. World Neurosurg..

[18] Zhu Z., Xu L., Jiang L., Sun X., Qiao J., Qian B.-P., Mao S., Qiu Y. (2017). Is Brace Treatment Appropriate for Adolescent Idiopathic Scoliosis Patients Refusing Surgery with Cobb Angle between 40 and 50 Degrees. Clin. Spine Surg..

[19] Negrini S., Negrini F., Fusco C., Zaina F. (2011). Idiopathic scoliosis patients with curves more than 45 Cobb degrees refusing surgery can be effectively treated through bracing with curve improvements. Spine J..

[20] Razeghinezhad R., Kamyab M., Babaee T., Ganjavian M.S., Bidari S. (2021). The Effect of Brace Treatment on Large Curves of 40° to 55° in Adolescents with Idiopathic Scoliosis Who Have Avoided Surgery: A Retrospective Cohort Study. Neurospine.

[21] Karavidas N. Complete Non-Operative Treatment with Brace and Scoliosis Specific Exercises Can Be Effective for Severe Scoliotic Curves Exceeding 40° at Peak of Growth. Proceedings of the SOSORT International Congress.

[22] Maruyama T., Kobayashi Y., Miura M., Nakao Y. (2014). Outcomes of brace treatment for adolescent idiopathic scoliosis with curve magnitude of 41 to 50 degrees. Scoliosis.

[23] Aulisa A.G., Guzzanti V., Falciglia F., Giordano M., Galli M., Aulisa L. (2019). Brace treatment of Idiopathic Scoliosis is effective for a curve over 40 degrees, but is the evaluation of Cobb angle the only parameter for the indication of treatment?. Eur. J. Phys. Rehabil. Med..

[24] Lusini M., Donzelli S., Minnella S., Zaina F., Negrini S. (2013). Brace treatment is effective in idiopathic scoliosis over 45°: An observational prospective cohort controlled study. Spine J..

[25] Bunge E.M., Habbema J.D.F., de Koning H.J. (2010). A Randomised Controlled Trial on the Effectiveness of Bracing Patients with Idiopathic Scoliosis: Failure to Include Patients and Lessons to Be Learnt. Eur. Spine J..

[26] Weinstein S.L., Dolan L.A., Wright J.G., Dobbs M.B. (2013). Effects of Bracing in Adolescents with Idiopathic Scoliosis. N. Engl. J. Med..

[27] Shaughnessy W.J. (2007). Advances in Scoliosis Brace Treatment for Adolescent Idiopathic Scoliosis. Orthop. Clin. North Am..

[28] Negrini S., Di Felice F., Negrini F., Rebagliati G., Zaina F., Donzelli S. (2022). Predicting final results of brace treatment of adolescents with idiopathic scoliosis: First out-of-brace radiograph is better than in-brace radiograph—SOSORT 2020 award winner. Eur. Spine J..

[29] Emans J.B., Kaelin A., Bancel P., Hall J.E., Miller M.E. (1986). The Boston Bracing System for Idiopathic Scoliosis. Follow-up Results in 295 Patients. Spine.

[30] Edmonsson A.S., Morris J.T. (1977). Follow-up study of Milwaukee brace treatment in patients with idiopathic scoliosis. Clin. Orthop. Relat. Res..

[31] Lange J.E., Steen H., Gunderson R., Brox J.I. (2011). Long-term results after Boston brace treatment in late-onset juvenile and adolescent idiopathic scoliosis. Scoliosis.

[32] Danielsson A.J., Hallerman K.L. (2015). Quality of Life in Middle-Aged Patients with Idiopathic Scoliosis with Onset before the Age of 10 Years. Spine Deform..

[33] Schwieger T., Campo S., Weinsteiny S.L., Dolan L.A., Ashida S., Steuber K.R. (2016). Body Image and Quality-of-Life in Untreated versus Brace-Treated Females with Adolescent Idiopathic Scoliosis. Spine.

[34] Tavernaro M., Pellegrini A., Tessadri F., Zaina F., Zonta A., Negrini S. (2012). Team care to cure adolescents with braces (avoiding low quality of life, pain and bad compliance): A case-control retrospective study. 2011 SOSORT Award winner. Scoliosis.

[35] Al-Mohrej O.A., Aldakhil S.S., Al-Rabiah M.A., Al-Rabiah A.M. (2020). Surgical treatment of adolescent idiopathic scoliosis: Complications. Ann. Med. Surg..

